# Identification of the 3-lncRNA Signature as a Prognostic Biomarker for Colorectal Cancer

**DOI:** 10.3390/ijms21249359

**Published:** 2020-12-08

**Authors:** Shuzhen Liu, Qing Cao, Guoyan An, Bianbian Yan, Lei Lei

**Affiliations:** Key Laboratory of Resource Biology and Biotechnology in Western China, Ministry of Education, School of Medicine, Northwest University, Xi’an 710069, China; LSZ15709483140@163.com (S.L.); caoqing140508028@163.com (Q.C.); 15291032749@163.com (G.A.); bbyan2018@163.com (B.Y.)

**Keywords:** competing endogenous RNA, long noncoding RNA, colorectal cancer

## Abstract

Colorectal cancer (CRC) is one of the most common malignant carcinomas in the world, and metastasis is the main cause of CRC-related death. However, the molecular network involved in CRC metastasis remains poorly understood. Long noncoding RNA (lncRNA) plays a vital role in tumorigenesis and may act as a competing endogenous RNA (ceRNA) to affect the expression of mRNA by suppressing miRNA function. In this study, we identified 628 mRNAs, 144 lncRNAs, and 25 miRNAs that are differentially expressed (DE) in metastatic CRC patients compared with nonmetastatic CRC patients from the Cancer Genome Atlas (TCGA) database. Functional enrichment analyses confirmed that the identified DE mRNAs are extensively involved in CRC tumorigenesis and migration. By bioinformatics analysis, we constructed a metastasis-associated ceRNA network for CRC that includes 28 mRNAs, 12 lncRNAs, and 15 miRNAs. We then performed multivariate Cox regression analysis on the ceRNA-related DE lncRNAs and identified a 3-lncRNA signature (LINC00114, LINC00261, and HOTAIR) with the greatest prognostic value for CRC. Clinical feature analysis and functional enrichment analysis further proved that these three lncRNAs are involved in CRC tumorigenesis. Finally, we used Transwell, Cell Counting Kit (CCK)-8, and colony formation assays to clarify that the inhibition of LINC00114 promotes the migratory, invasive, and proliferative abilities of CRC cells. The results of the luciferase assay suggest that LINC00114 is the direct target of miR-135a, which also verified the ceRNA network. In summary, this study provides a metastasis-associated ceRNA network for CRC and suggests that the 3-lncRNA signature may be a useful candidate for the diagnosis and prognosis of CRC.

## 1. Introduction

Worldwide, the incidence of colorectal cancer (CRC) ranks third and the mortality rate ranks second. Despite a decreasing trend of CRC mortality in many developed countries, the highest CRC incidence rates occur in Europe, Australia, and Northern America. Moreover, some developing countries have shown increases in both incidence and mortality in recent decades [1]. Metastasis is the main cause of CRC-related death, but the molecular mechanisms remain poorly understood [2]. Therefore, investigating the molecular networks that regulate metastasis is imperative for improving CRC early diagnosis and therapy.

Except for a few transcripts with protein-coding capacity, the vast majority of transcripts in humans are noncoding RNAs, which comprise two classes: small noncoding RNAs (sncRNAs) and long noncoding RNAs (lncRNAs) [3,4]. MicroRNAs (miRNAs) are sncRNAs with a length of approximately 21–24 nucleotides (nt) that target the 3′ untranslated region of mRNAs to suppress gene expression [5,6]. In contrast, lncRNAs are greater than 200 nt in length, act as translational regulators to regulate the degradation and stability of mRNAs, and play critical roles in the progression of many cancers, including CRC [7,8,9]. For instance, high expression levels of MALAT1, HOTAIR, and H19 correlate tightly with metastasis and a poor prognosis in CRC patients [10,11,12]. Salmena et al. reported that lncRNAs can act as endogenous “miRNA sponges” to inhibit miRNA function by sharing miRNA response elements (MREs) [13]. Increasing evidence indicates that molecular networks play important roles in a wide variety of human diseases [14]. Dysregulation of the competing endogenous RNA (ceRNA) network could promote tumorigenesis, including CRC [15,16].

The biological function of lncRNAs and the ceRNA network in CRC have been characterized. A recent study demonstrated that the lncRNAs KCNQ1OT1 and WT1-AS interact with most of the miRNAs in the ceRNA network and, thus, may play important roles in CRC progression [17]. Moreover, LINC00400 and LINC00355 contribute to the regulation of the ceRNA network in CRC [18]. In this study, we aim to identify a lncRNA signature as a biomarker for the diagnosis and prognosis of CRC. We first applied bioinformatics methods to construct a metastasis-associated ceRNA network. Then, multivariate Cox regression analysis was used to identify a 3-lncRNA signature according to CRC patient survival. Furthermore, we verified the function of LINC00114 using Transwell, CCK-8, and colony formation assays with CRC cell lines. This study reveals a metastasis-associated ceRNA network in CRC that might provide useful candidates for CRC pathogenesis.

## 2. Results

### 2.1. Metastasis-Specific mRNAs, miRNAs, and lncRNAs in CRC Patients

**Figure 1:**
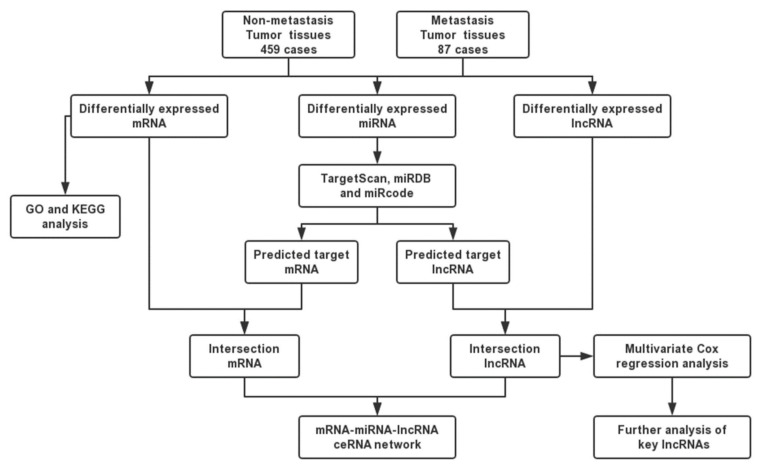
Flow chart of construction of competing endogenous RNA (ceRNA) network.

To investigate metastasis-specific mRNAs, miRNAs, and lncRNAs in CRC tissues, we used RNA expression profiles from the TCGA dataset, including 459 nonmetastatic CRC samples and 87 metastatic CRC samples (Figure 1). The results revealed 628 DE mRNAs between nonmetastatic and metastatic colorectal tumor tissues, of which 354 were upregulated and 274 were downregulated. We also obtained 144 dysregulated lncRNAs and 25 aberrantly expressed miRNAs using the “edgeR” package in R.

### 2.2. Functional Enrichment Analysis of DE mRNA

**Figure 2:**
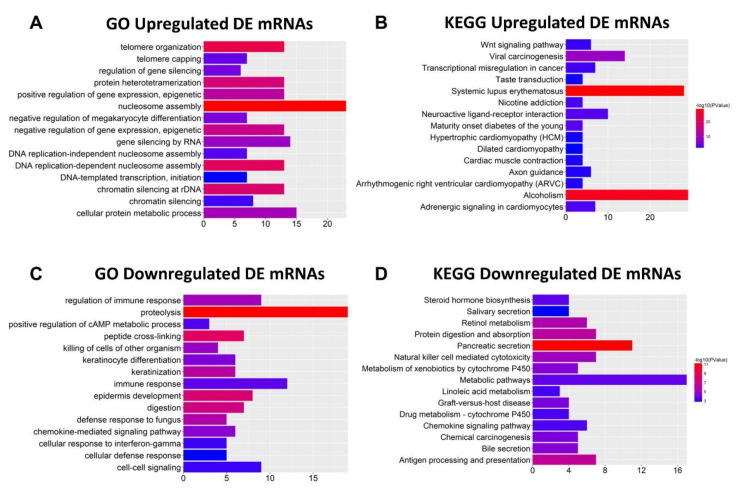
Functions of differentially expressed (DE) mRNAs were analyzed with DAVID and KOBAS bioinformatics resources, respectively. (**A**,**B**) Top 15 Gene Ontology (GO) and Kyoto Encyclopedia of Genes and Genomes (KEGG) results of upregulated mRNAs, respectively. (**C**,**D**) Top 15 GO and KEGG results of downregulated mRNAs, respectively. Color from blue to red indicates −log10 (*p*) from low to high. *X*-axes represent the number of genes involved in each pathway.

To identify the functions of the DE mRNAs, the 628 dysregulated mRNAs were divided into two groups (354 upregulated and 274 downregulated) for Gene Ontology (GO) and Kyoto Encyclopedia of Genes and Genomes (KEGG) pathway analyses using DAVID and KOBAS bioinformatics resources, respectively (Figure 2). The top 15 GO and KEGG pathways indicated that the upregulated genes participate in “GO:0045815~positive regulation of gene expression, epigenetic”, “GO:0006342~chromatin silencing”, “Transcriptional misregulation in cancer”, and “Wnt signaling pathway”, which are considered to be associated with cancer metastasis [19,20,21,22]. In addition, “GO:0032200~telomere organization” and “Viral carcinogenesis” are related to oncogenesis [23]. Among the top 15 GO and KEGG pathways of downregulated mRNAs, “GO:0008544~epidermis development”, “GO:0030216~keratinocyte differentiation”, “Natural killer cell-mediated cytotoxicity”, “Chemical carcinogenesis”, and “Metabolism of xenobiotics by cytochrome P450” are related to tumorigenesis and cancer cell migration [24,25,26,27]. These findings indicate that these DE mRNAs might be associated with CRC metastasis.

### 2.3. Construction of the ceRNA Network

MiRNAs play a major role in ceRNA networks by suppressing the expression of target mRNAs. Salmena et al. reported that lncRNAs could act as miRNA sponges by occupying their binding sites and inhibiting their functions [13]. In this study, we used miRDB and Targetscan to predict target mRNAs of 25 DE miRNAs. We then compared the predicted mRNAs with 628 DE mRNAs and selected 28 mRNAs to construct the ceRNA network (Table S1). We also studied whether these 25 miRNAs target 144 DE lncRNAs. According to the miRcode results, 9 miRNAs are predicted to interact with 12 lncRNAs (Table S2).

**Figure 3:**
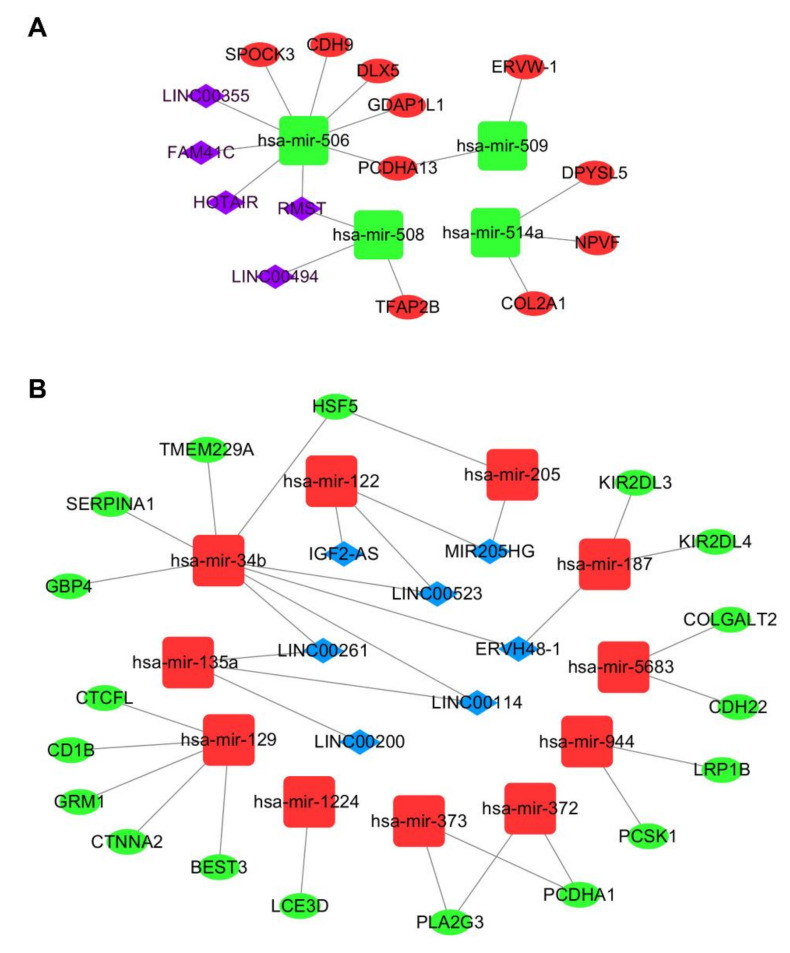
Metastasis-associated ceRNA network for colorectal cancer (CRC). (**A**) Green squares represent downregulated miRNAs, red ellipses represent upregulated mRNAs, and purple diamonds represent upregulated lncRNAs. (**B**) Red squares represent upregulated miRNAs, green ellipses represent downregulated mRNAs, and blue diamonds represent downregulated lncRNAs.

Based on the above evidence (Tables S1 and S2), we next constructed a ceRNA network using Cytoscape v3.7. Ultimately, 28 mRNAs, 15 miRNAs (Table S3), and 12 lncRNAs (Table S4) are involved in the ceRNA network. Figure 3 shows that 4 downregulated miRNAs, 10 upregulated mRNAs, and 5 upregulated lncRNAs are involved in one ceRNA network. Meanwhile, 11 upregulated miRNAs, 18 downregulated mRNAs, and 7 downregulated lncRNAs are involved in another ceRNA network.

### 2.4. Construction of the 3-lncRNA Prognostic Model

**Figure 4:**
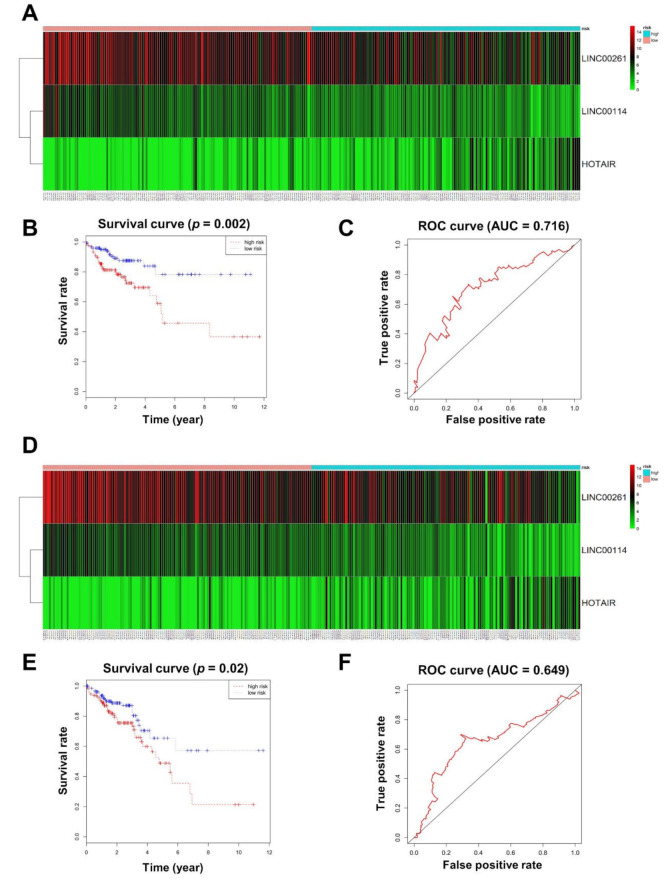
Prognostic evaluation of the 3-lncRNA (long noncoding RNA) signature. (**A**) CRC patients in the training set were divided into high- and low-risk groups using the risk score generated from the 3-lncRNA signature. The heatmap shows the expression profiles of the 3 lncRNAs in CRC patients. Color from green to red indicates an expression level from low to high, respectively. (**B**) The Kaplan–Meier curves of the 3-lncRNA signature for overall survival among CRC patients in the training set (*p* = 0.002). (**C**) Receiver operating characteristic (ROC) curve for predicting 5-year survival in CRC patients in the training set based on risk scores. (**D**) CRC patients in the testing set were divided into high- and low-risk groups using the risk score generated from the 3-lncRNA signature. (**E**) The Kaplan–Meier curves of the 3-lncRNA signature for overall survival among CRC patients in the testing set (*p* = 0.02). (**F**) ROC curve for predicting 5-year survival in CRC patients in the testing set based on risk scores.

The 12 lncRNAs in the ceRNA network were further analyzed according to CRC patient survival. We randomly divided CRC patients into a training group (*n* = 272) and a testing group (*n* = 272), and no significant differences were found between the two groups (Table S5). By multivariate Cox regression analysis, three lncRNAs (LINC00114, LINC00261, and HOTAIR) displayed a remarkable prognostic value in the training set (Table S6). HOTAIR was associated with high risk, whereas LINC00114 and LINC00261 were protective (Figure 4A). CRC patients were classified into low-risk (*n* = 136) and high-risk (*n* = 136) sets based on the risk score formula (risk score = 0.1184 × HOTAIR − 0.2257 × LINC00114 − 0.1477 × LINC00261), and Kaplan–Meier analysis indicated a positive correlation with overall survival (OS) for the low-risk group compared with the high-risk group (*p* = 0.002; Figure 4B). Additionally, the accuracy of the prognostic model was evaluated by receiver operating characteristic (ROC) curve analysis, and the area under the ROC curve (AUC) at 5 years for OS was 0.716 (Figure 4C).

In order to verify the predictive ability of the 3-lncRNA model, Kaplan–Meier analysis and ROC curve analyses were further used on the testing group. According to the previous risk score formula, patients in the testing group were divided into low-risk and high-risk groups (Figure 4D). There were statistically significant differences between the low-risk group and the high-risk group in both Kaplan–Meier analysis (*p* = 0.02; Figure 4E) and ROC curve analysis (AUC = 0.649; Figure 4F). These findings demonstrate that the 3-lncRNA signature may act as an independent biomarker that predicts CRC survival prognosis.

### 2.5. Clinical Feature Analysis of the Key lncRNAs

**Figure 5:**
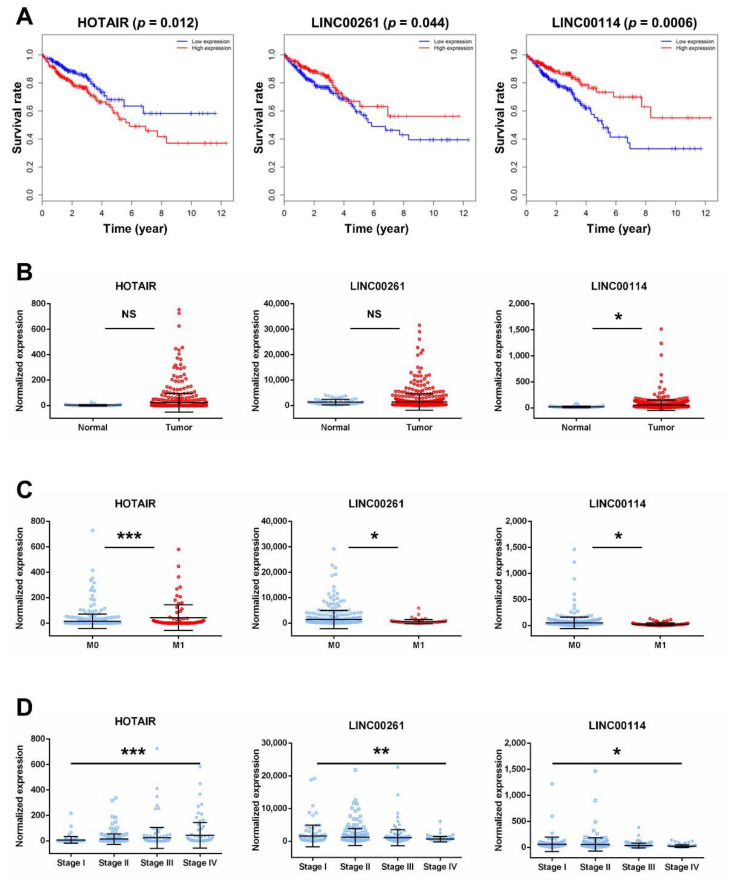
Clinical feature analysis of HOTAIR, LINC00261, and LINC00114. (**A**) Kaplan–Meier analysis of CRC overall survival according to the three lncRNAs. Percent survival is shown on the *y*-axis, and time is shown on the *x*-axis. Red lines represent patients with higher expression levels of lncRNAs, and blue lines represent patients with lower expression levels of lncRNAs (*p* < 0.05 log-rank test). (**B**) LncRNA expression analysis in CRC tissues and normal tissues. (**C**) LncRNA expression analysis in metastatic CRC tissues (M1) and nonmetastatic samples (M0). (**D**) LncRNA expression analysis in stages 1–4 of CRC. Significant differences were assessed by nonpaired *t*-tests. n.s., not significant; * *p* < 0.05, ** *p* < 0.01, *** *p* < 0.001.

To further research the three lncRNAs, we analyzed the association between the expression of lncRNAs and clinical features of CRC. Kaplan–Meier survival curves indicated that HOTAIR was negatively correlated with OS (*p* = 0.012), while LINC00261 and LINC00114 were positively correlated with OS (*p* = 0.044, *p* = 0.0006, respectively; Figure 5A). Although there was no significant difference in the expression of HOTAIR in normal tissues and tumors (Figure 5B), HOTAIR was upregulated in metastatic CRC tissues and stage 4 tissues compared with nonmetastatic samples and stage 1 tissues, respectively (Figure 5C,D). In contrast, the expression of LINC00261 showed no significance between normal tissues and tumors and was downregulated in metastatic CRC tissues and stage 4 tissues (Figure 5B–D). The expression of LINC00114 was downregulated in tumor tissues, metastatic CRC tissues, and stage 4 tissues (Figure 5B–D).

### 2.6. Functional Enrichment Analysis of the Key lncRNAs

We further investigated the function of the three key lncRNAs according to the principle that coexpressed genes might share roles in related biological processes [28,29]. First, we searched for mRNAs that are coexpressed with the key lncRNAs based on the web resource Multi-Experiment Matrix (MEM) [30], which searches a large number of microarray datasets and uses rank aggregation to detect globally coexpressed genes. The top 200 mRNAs that are potentially coexpressed with HOTAIR and LINC00261 are shown in Table S7, while LINC00114 could not be searched for on MEM.

**Figure 6:**
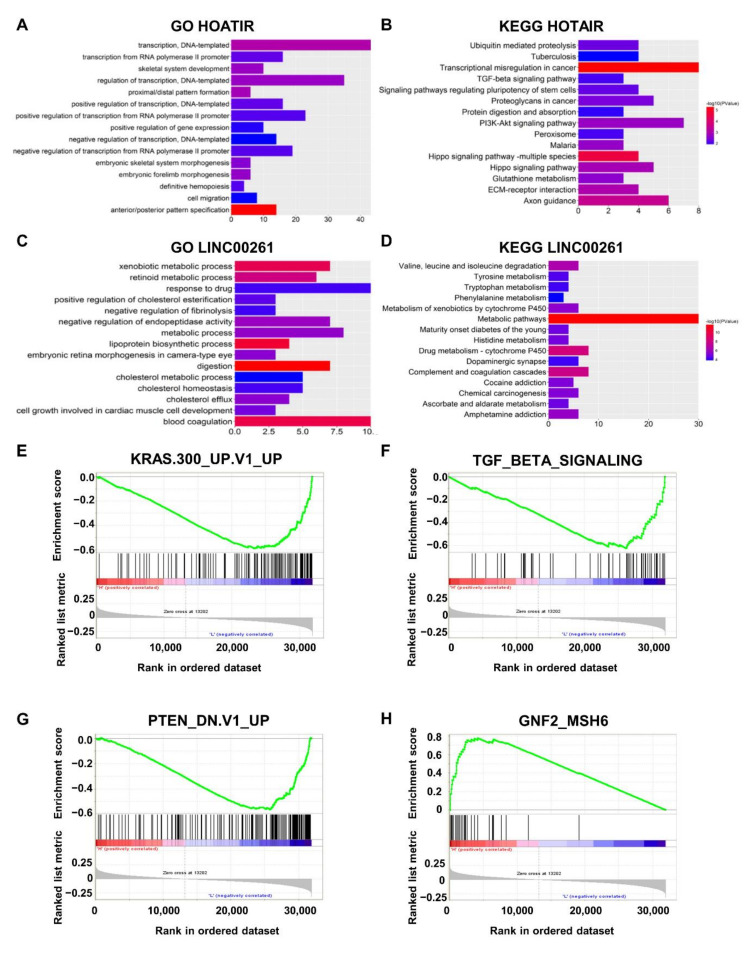
Enrichment analysis of HOTAIR, LINC00261, and LINC00114. (**A**,**B**) Top 15 GO and KEGG results for coexpressed genes of HOTAIR. (**C**,**D**) Top 15 GO and KEGG results of the coexpressed genes of LINC00261. Color from blue to red indicates −log10 (*p*) from low to high. *X*-axes represent the number of genes involved in each pathway. (**E**,**F**) The gene sets “KRAS.300_UP.V1_UP” (*p* < 0.0001) and “TGF_BETA_SIGNALING” (*p* = 0.002) were significantly enriched with low levels of LINC00114. (**G**) The gene sets “PTEN_DN.V1_UP” (*p* < 0.0001), which included genes upregulated upon knockdown of PTEN, were significantly enriched with low levels of LINC00114. (**H**) The gene set “GNF2_MSH6” (*p* = 0.018) was enriched with high levels of LINC00114.

We then used GO and KEGG pathway enrichment analyses to assess the enrichment of these mRNAs. The top 15 biological processes in the GO and KEGG pathways showed that HOTAIR participates in “GO:0016477~cell migration”, “Hippo signaling pathway”, “Ubiquitin-mediated proteolysis”, and “TGF-beta signaling pathway” (Figure 6A,B), which are correlated with tumorigenesis and cancer metastasis [31,32,33]. The mRNAs positively coexpressed with LINC00261 are mainly involved in “GO:0042158~lipoprotein biosynthetic process”, “GO:0008152~metabolic process”, “Drug metabolism—cytochrome P450”, and “Chemical carcinogenesis” (Figure 6C,D), suggesting that LINC00261 may regulate tumorigenesis [24,34] and also coincide with functional enrichment of metastasis-associated downregulated mRNAs (Figure 2D).

We performed GSEA to further determine the pathways in which LINC00114 affects CRC. The gene sets “KRAS.300_UP.V1_UP” (*p* < 0.0001), “TGF_BETA_SIGNALING” (*p* = 0.002), and “PTEN_DN.V1_UP” (*p* < 0.0001) were remarkably enriched with low levels of LINC00114, while “GNF2_MSH6” (*p* = 0.018) was enriched with high levels of LINC00114 (Figure 6E–H). Many studies have shown that both activation of KRAS [35] and TGF-β pathways [36] and loss of PTEN [37] and MSH6 [38] are the driving force of tumorigenesis [39]. Therefore, these results suggest that LINC00114 might be involved in CRC progression as a tumor suppressor.

### 2.7. LINC00114 Represses CRC Cell Migration and Proliferation

**Figure 7:**
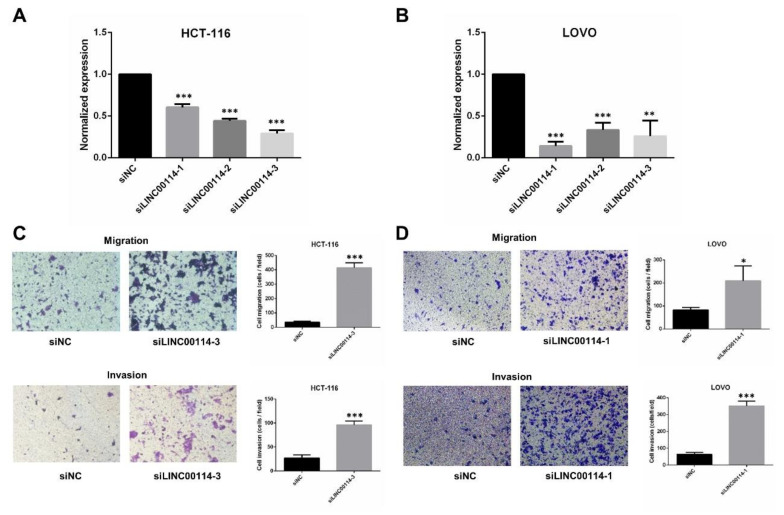
LINC00114 represses CRC cell migration. (**A**,**B**) LINC00114 expression levels were determined in negative control or siRNA-transfected HCT-116 or LOVO cells by qRT-PCR. Expression levels were normalized to *GAPDH*, and the results were expressed as the fold increase relative to negative control. (**C**,**D**) Transwell assays were used to determine the role of LINC00114 siRNA in HCT-116 or LOVO cell migration and invasion. All data are expressed as mean ± standard deviation (SD) of three independent experiments; * *p* < 0.05, ** *p* < 0.01, *** *p* < 0.001.

**Figure 8:**
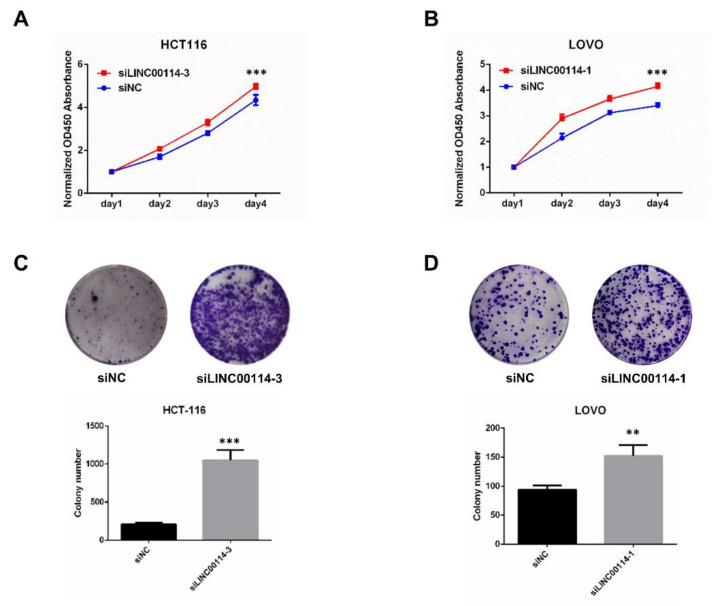
LINC00114 represses CRC cell proliferation. (**A**–**D**). Cell Counting Kit (CCK)-8 and colony formation assays were used to assess the role of LINC00114 siRNA in HCT-116 or LOVO cell proliferation. All data are expressed as mean ± SD of three independent experiments; ** *p* < 0.01, *** *p* < 0.001.

To further determine whether LINC00114 impacts CRC tumorigenesis, we validated the effect of LINC00114 knockdown on the migration and proliferation of CRC cells. After we confirmed the suppression efficiency of the three LINC00114 siRNAs in HCT-116 and LOVO cells by quantitative RT-PCR, siLINC00114-3 and siLINC00114-1 were selected for subsequent functional experiments, respectively (Figure 7A,B). Transwell assays showed that compared to the negative control, suppression of LINC00114 by siRNA promoted both migration and invasion abilities (Figure 7C,D). CCK-8 and colony formation assays were used to assess the role of LINC00114 in CRC proliferation. Compared to the negative control, inhibition of LINC00114 significantly promoted CRC cell proliferation (Figure 8A–D). These results indicate that LINC00114 represses the migratory, invasive, and proliferative abilities of CRC cells.

**Figure 9:**
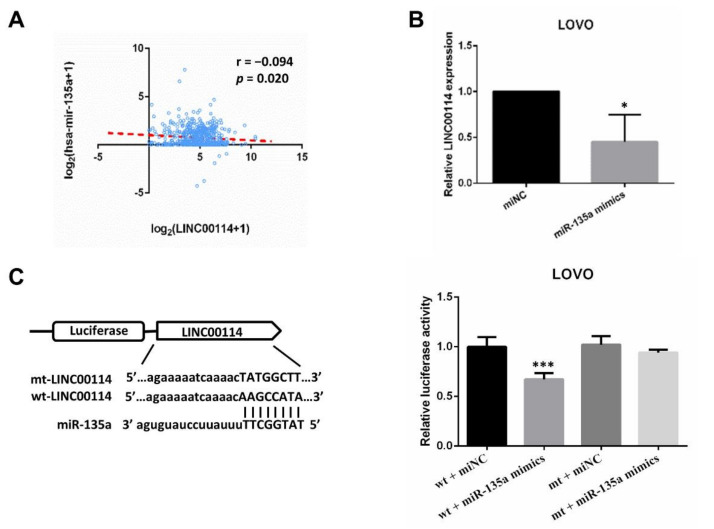
LINC00114 is the direct target of miR-135a. (**A**) Pearson’s correlation analysis showed that miR-135a was negatively associated with LINC00114. (**B**) The levels of LINC00114 were determined in negative control or miR-135a-mimic-transfected LOVO cells by qPCR. (**C**) Schematic illustration shows the miR-135a binding site and the reverse complementary sequence mutations in LINC00114. Luciferase assay was used to identify the miR-135a binding site. All data are expressed as mean ± SD of three independent experiments; * *p* < 0.05, *** *p* < 0.001.

The ceRNA networks revealed that LINC00114 may target hsa-mir-34b and hsa-mir-135a. By Pearson correlation analysis, we found that LINC00114 was negatively associated with hsa-mir-135a (Figure 9A). This result was also confirmed by transfecting miR-135a mimics in LOVO cells, which indicated that overexpression of miR-135a suppressed the expression of LINC00114 (Figure 9B). To figure out whether LINC00114 is a direct target of miR-135a, we constructed plasmids with a wild-type fragment of LINC00114 (wt-LINC00114) and a mutant fragment of LINC00114 (mt-LINC00114). Luciferase assays showed that coexpression of miR-135a mimics luciferase activity and wt-LINC00114 markedly reduced luciferase activity compared with the control (Figure 9C). These data suggest that LINC00114 is the direct target of miR-135a, which also verifies the ceRNA network.

## 3. Discussion

CRC is one of the most common tumors worldwide, and it was the second leading cause of mortality in 2018 [1]. Metastasis is the main reason for the poor overall 5-year survival rate of advanced cases [2]. Recently, an increasing number of articles have shown that dysregulated lncRNA expression is related to CRC tumorigenesis and metastasis [10,11,12]. The ceRNA network hypothesis suggests that lncRNAs are able to regulate mRNA expression as endogenous “miRNA sponges” by binding to miRNA MREs [13]. However, the role of ceRNAs in CRC metastasis has remained unclear. The present study reveals the metastasis-associated ceRNA network in CRC, which provides new biomarkers for predicting CRC survival.

In this study, we identified DE mRNAs, lncRNAs, and miRNAs between metastatic CRC patients and nonmetastatic CRC patients. We used bioinformatics methods to construct a metastasis-associated ceRNA network that includes 28 mRNAs, 15 miRNAs, and 12 lncRNAs. By using the MIENTURNET web tool, we found that most of the DE miRNAs are remarkably enriched in pathways associated with tumorigenesis [40]. The results of GO and KEGG pathway analyses indicated that upregulated mRNAs mainly participate in “Transcriptional misregulation in cancer” and “Wnt signaling pathway”. Many studies have shown that invasive edge-localized CRC cells exhibit strong nuclear β-catenin staining [41] and preferentially express Wnt target genes, which confer invasive metastatic capacity [42,43]. In contrast, downregulated mRNAs were enriched in “GO:0008544~epidermis development” and “GO:0030216~keratinocyte differentiation”, in which small proline-rich protein 2F (*SPRR2F*), small proline-rich protein 1B (*SPRR1B*), small proline-rich protein 2G (*SPRR2G*), late cornified envelope 3D (*LCE3D*), and late cornified envelope 1E (*LCE1E*) are involved. A previous study showed that overexpression of SPRR proteins increases local aggressiveness but prevents metastasis in cholangiocarcinoma [44]. Therefore, the downregulation of these genes promotes the metastatic capacity of CRC. These data indicate that the metastasis-related DE mRNAs are involved in CRC tumorigenesis and migration.

LncRNAs play critical roles in the progression of cancers and may act as candidate indicators to predict survival prognosis. A previous study showed that a 4-lncRNA signature (LINC01555, RP11-610P16.1, RP11-108K3.1, and LINC01207) had the most significant correlation with OS in colon adenocarcinoma patients [45]. In this study, we determined dysregulated lncRNA profiles in metastatic CRC and constructed metastasis-associated ceRNA networks in large CRC cohorts. We further found that 3 lncRNAs (LINC00114, LINC00261, and HOTAIR) showed a significant prognostic value for CRC based on multivariate Cox regression analysis. HOTAIR has been reported to increase in primary tumors and metastases, and its expression level is a powerful predictor of metastasis and mortality [10,20,46,47,48]. Additionally, a recent study showed that HOTAIR controls Rab22a expression in ovarian cancer by sponging miR-373 [49] and regulates HER2 expression in gastric cancer by sponging miR-331-3p [50]. In our study, HOTAIR was found to be negatively associated with OS and was upregulated in metastatic CRC and stage 4 disease. Moreover, GO and KEGG pathway analyses showed that the mRNAs that are positively coexpressed with HOTAIR participate in “GO:0016477~cell migration”, “Transcriptional misregulation in cancer”, “Hippo signalling pathway”, and “TGF-beta signaling pathway”, which are considered to be correlated with CRC tumorigenesis and metastasis [31,32,33].

LINC00261 also plays a notable role in CRC pathogenesis and metastasis. Recent research has demonstrated that overexpression of LINC00261 relieves cisplatin resistance in CRC cells by promoting apoptosis and inhibiting migration [51]. Several studies have shown that LINC00261 exerts tumor-suppressive effects in gastric cancer and pancreatic cancer [52,53]. Moreover, LINC00261 competes with miR-132-3p to regulate the expression of BCL2L11 in endometriosis [54]. In the present study, LINC00261 was positively correlated with OS and downregulated in metastatic CRC tissues and stage 4 disease. Interestingly, LINC00261 coexpressed genes were mainly clustered in “GO:0008152~metabolic process”, “Drug metabolism—cytochrome P450”, and “Chemical carcinogenesis”, which also coincided with the functional enrichment of metastasis-associated downregulated mRNAs. These results indicate that overexpression of LINC00261 may inhibit CRC tumorigenesis and metastasis.

We used Transwell, CCK-8, and colony formation assays to verify the function of LINC00114 in CRC cell lines. The results indicated that suppression of LINC00114 promoted migratory, invasive, and proliferative abilities compared to the negative control, which was consistent with in-silico analysis. However, a recent study showed that LINC00114 depletion inhibited CRC cell proliferation [55]. As they did not investigate the role of LINC00114 in the migration of CRC cells, we will further study the functional mechanism of LINC00114 in the future.

Based on the ceRNA network, LINC00114 may compete with two key DE miRNAs (miR-135a and miR-34b) to regulate the expression of target genes. Several studies have confirmed that miR-135a and miR-34b are highly expressed in CRC tissues and promote CRC cell proliferation, migration, and invasion [56,57,58]. In this article, we showed that LINC00114 is the direct target of miR-135a by luciferase assay.

In conclusion, we constructed a metastasis-associated ceRNA network in CRC and proved that a 3-lncRNA signature that includes LINC00114, LINC00261, and HOTAIR is an independent factor for predicting CRC prognosis. The current study provides insight into the identification of potential biomarkers for the diagnosis and prognosis of CRC.

## 4. Materials and Methods

### 4.1. Study Population and Identification of Differential Expression

CRC transcriptome profiles (level 3) and corresponding clinical information were downloaded. These data included 51 normal tissues and 649 CRC tissues. According to the TNM staging systems, the number of M1 (metastatic cases) and M0 (nonmetastatic cases) tumor tissues were 87 and 459, respectively (unknown information for 103 tumor tissues).

We divided the CRC samples into 2 groups (M0 and M1) and used the “edgeR” package in R software to analyze DE RNAs (mRNA, miRNA, and lncRNA) [59]. Compared to the M0 group, we obtained 628 DE mRNAs, 144 DE lncRNAs, and 25 DE miRNAs, with thresholds of |logFC| > 1 and FDR (false discovery rate) < 0.05.

### 4.2. Functional Enrichment Analysis

To explore the functions of the identified DE mRNAs, 628 DE mRNAs were divided into an upregulated group and a downregulated group for Gene Ontology (GO) analysis and Kyoto Encyclopedia of Genes and Genomes (KEGG) pathway analysis. DAVID was used to analyze GO biological processes, and KOBAS was used to analyze KEGG pathways. The top 15 pathways were visualized using the R package ggplot2.

To clarify the function of the DE lncRNAs, we first searched for coexpressed mRNAs based on the web resource Multi-Experiment Matrix (MEM). The top 200 potentially coexpressed mRNAs for HOTAIR and LINC00261 were selected for GO and KEGG analyses.

Gene set enrichment analysis (GSEA) was performed to investigate the function of LINC00114. CRC gene sets were obtained from the TCGA database and uploaded to GSEA software (v3.0). The phenotype label was high-level of LINC00114 versus low-level of LINC00114. The permutation number was 1000, and a ranked-list metric was created by counting the signal-to-noise ratio.

### 4.3. Construction of the ceRNA Network

We used metastasis-associated DE mRNAs, lncRNAs, and miRNAs to construct the ceRNA network. miRDB and Targetscan were used to predict mRNA-miRNA interactions. In addition, miRcode was performed to construct lncRNA–miRNA interactions. We retained intersections with the DE mRNAs, miRNAs, and lncRNAs to build the ceRNA network, as the flow chart in Figure 1 illustrates. Cytoscape v3.7.1 was used to visualize the ceRNA network.

### 4.4. Definition of the Prognostic Model and the Survival Curve

The 544 CRC patients were randomly divided into a training group (*n* = 272) and a testing group (*n* = 272). Multivariate Cox regression analysis was performed to identify the prognostic model for the lncRNAs in the ceRNA network. Based on the Akaike information criterion (AIC), the best mathematical model was established using the “survival” R package [60]. A risk score for predicting overall survival (OS) was generated by including the expression level of each lncRNA (explncRNA) weighted by the regression coefficient (βlncRNA), as follows:

Risk score = βlncRNA1 × explncRNA1 + βlncRNA2 × explncRNA2 + βlncRNAn × explncRNAn.

According to the risk score of the lncRNA prognostic model, patients were divided into a low-risk group and a high-risk group. Kaplan–Meier analysis was utilized to generate the OS curve for the two groups. The accuracy of the prognostic model within 5 years was evaluated by time-dependent receiver operating characteristic (ROC) curve analysis using the “survivalROC” R package [61].

### 4.5. Cell Lines and Transfection

HCT-116 and LOVO human CRC cells were purchased from American Type Culture Collection (ATCC). A short interfering RNA (siRNA) for LINC00114 and a negative control were transfected at 100 nM according to the instructions for the X-tremeGENE siRNA transfection reagent (Roche, Mannheim, Germany). The following siRNA sequences were used in this study: siLINC00114-1, sense, 5′-GGAGCCGACUUGCUUAGAATT-3′; antisense: 5′-UUCUAAGCAAGUCGGCUCCTT-3′; siLINC00114-2, sense, 5′-GCGCAUCUCCUAAGAACUUTT-3′, antisense, 5′-AAGUUCUUAGGAGAUGCGCTT-3′; siLINC00114-3, sense, 5′-GCAUGAAGACUAAAUCCAUTT-3′, antisense: 5′-AUGGAUUUAGUCUUCAUGCTT-3′.

### 4.6. RNA Extraction and Quantitative RT-PCR

Total RNA was extracted from transfected HCT-116 cells using TRIzol (Invitrogen, Carlsbad, CA, USA). After cDNA synthesis (TAKARA, Dalian, China), quantitative real-time PCR assays were performed using the SYBR Premix Ex Taq™ II kit (TAKARA, Dalian, China) with a CFX96 Touch Real-Time PCR Detection System (Bio-Rad, Hercules, CA, USA). The following primer sequences were used in this study: LINC00114, forward, 5′-ACTTGTGGCATCATAGCAG-3′, reverse, 5′-AAGTGAAACAGTTCCCTTCT-3′; GAPDH, forward, 5′-TGGAAATCCCATCACCATCT-3′, reverse, 5′-TGGACTCCACGACGTACTCA-3′.

### 4.7. Transwell Assays

Transwell assays (Corning, MA, USA), without or with Matrigel (BD Biosciences, Bedford, MA, USA), were used to determine cell migration or invasion ability, respectively. Briefly, 5 × 104 HCT-116 or LOVO cells with serum-free medium were placed into the upper chamber, with serum-containing medium in the bottom chamber. After 48 h, the migrated cells were fixed in 4% paraformaldehyde and then stained with crystal violet.

### 4.8. Cell Proliferation

After 48 h of transfection, HCT-116 or LOVO cells were seeded at a density of 1000 cells per well into 96-well plates, with 6 replicates. Briefly, 10% of Cell Counting Kit (CCK)-8 solution (Dojindo, Kyushu, Japan) was incubated at 37 °C for 3 h and measured at a wavelength of 450 nm.

For colony formation assays, we seeded 1000 transfected HCT-116 or LOVO cells per well in 6-well plates. Fourteen days later, the number of colonies was counted. Each assay was performed in triplicate.

### 4.9. Dual-Luciferase Assay

The LINC00114 wild-type fragment (wt-LINC00114) and mutant fragment (mt-LINC00114) were constructed in the pmiRGLO plasmid (Promega, Madison, WI, USA). For dual-luciferase assay, the method has been described previously [62].

### 4.10. Statistical Analysis

GraphPad Prism 6 (GraphPad Software Inc., La Jolla, CA, USA) was used for nonpaired t-tests and Pearson’s correlation analyses. R Studio (R version 3.3.2) was used for bioinformatics analyses.

## Supplementary Materials

Supplementary Materials can be found at https://www.mdpi.com/1422-0067/21/24/9359/s1. Table S1: 13 DE miRNAs target with 28 DE mRNAs in metastasis CRC; Table S2: 9 DE miRNAs interact with 12 DE lncRNAs in metastasis CRC; Table S3: Metastasis CRC specific DE miRNAs in ceRNA network; Table S4: Metastasis CRC specific DE lncRNAs in ceRNA network; Table S5: Clinical covariates in the training and testing sets; Table S6: 3-lncRNA risk score model; Table S7: The top 200 mRNAs co-expressed with LINC00261 and HOTAIR.
