# Supplementary material for: Identification of the 3-lncRNA Signature as a Prognostic Biomarker for Colorectal Cancer
Source: Int J Mol Sci. 2020 Dec 8;21(24):9359. doi: 10.3390/ijms21249359 (PMC7764807; doi:10.3390/ijms21249359)
Supplement: Supplementary file 1 [file ijms-21-09359-s001.pdf]

**Table S1** 13 DE miRNAs target with 28 DE mRNAs in metastasis CRC

| miRNA | mRNA |
|---|---|
| hsa-mir-1224 | LCE3D |
| hsa-mir-129 | BEST3, CD1B, CTNNA2, CTCFL, GRM1 |
| hsa-mir-187 | KIR2DL3, KIR2DL4 |
| hsa-mir-205 | HSF5 |
| hsa-mir-34b | HSF5, GBP4, SERPINA1, TMEM229A |
| hsa-mir-372 | PLA2G3, PCDHA1 |
| hsa-mir-373 | PLA2G3, PCDHA1 |
| hsa-mir-5683 | CDH22, COLGALT2 |
| hsa-mir-944 | PCSK1, LRP1B |
| hsa-mir-506 | CDH9, PCDHA13, SPOCK3, GDAP1L1, DLX5 |
| hsa-mir-508 | TFAP2B |
| hsa-mir-509 | ERVW-1, PCDHA13 |
| hsa-mir-514a | NPVF, COL2A1, DPYSL5 |

**Table S2** 9 DE miRNAs interact with 12 DE lncRNAs in metastasis CRC

| miRNA | lncRNA |
|---|---|
| hsa-mir-122 | IGF2-AS, LINC00523, MIR205HG |
| hsa-mir-135a | LINC00114, LINC00200, LINC00261 |
| hsa-mir-187 | ERVH48-1 |
| hsa-mir-205 | MIR205HG |
| hsa-mir-34b | LINC00523, LINC00114, ERVH48-1, LINC00261 |
| hsa-mir-372 | LINC00494 |
| hsa-mir-373 | LINC00494 |
| hsa-mir-506 | LINC00355, HOTAIR, FAM41C, RMST |
| hsa-mir-508 | LINC00494, RMST |

**Table S3** Metastasis CRC specific DE miRNAs in ceRNA network

| miRNAs | Regulation | logFC | PValue | FDR |
|---|---|---|---|---|
| hsa-mir-508 | Downregulation | -1.84533 | 2.95E-08 | 9.91E-07 |
| hsa-mir-514a | Downregulation | -1.66489 | 1.45E-05 | 0.000318 |
| hsa-mir-506 | Downregulation | -1.588 | 6.93E-05 | 0.001099 |
| hsa-mir-509 | Downregulation | -1.34209 | 4.61E-06 | 0.000125 |
| hsa-mir-34b | Upregulation | 1.094529 | 6.69E-11 | 3.19E-09 |
| hsa-mir-129 | Upregulation | 1.266638 | 2.93E-13 | 1.67E-11 |
| hsa-mir-187 | Upregulation | 1.584598 | 1.52E-11 | 7.89E-10 |
| hsa-mir-122 | Upregulation | 1.635523 | 1.31E-05 | 0.000299 |
| hsa-mir-1224 | Upregulation | 1.642063 | 3.01E-09 | 1.22E-07 |
| hsa-mir-5683 | Upregulation | 1.847101 | 3.34E-14 | 2.38E-12 |
| hsa-mir-944 | Upregulation | 2.051589 | 1.95E-20 | 1.59E-18 |
| hsa-mir-135a | Upregulation | 2.367054 | 9.11E-23 | 1.04E-20 |
| hsa-mir-205 | Upregulation | 2.38084 | 3.19E-09 | 1.22E-07 |
| hsa-mir-372 | Upregulation | 6.908926 | 1.52E-83 | 8.67E-81 |
| hsa-mir-373 | Upregulation | 7.415244 | 8.45E-68 | 2.41E-65 |

**Table S4** Metastasis CRC specific DE lncRNAs in ceRNA network

| lncRNAs | Regulation | logFC | PValue | FDR |
|---|---|---|---|---|
| LINC00200 | Downregulation | -2.43508 | 5.53E-05 | 0.00283 |
| MIR205HG | Downregulation | -1.96056 | 0.002168 | 0.041671 |
| ERVH48-1 | Downregulation | -1.84051 | 8.89E-08 | 1.48E-05 |
| LINC00523 | Downregulation | -1.69343 | 0.000632 | 0.017379 |
| IGF2-AS | Downregulation | -1.52588 | 2.49E-05 | 0.001514 |
| LINC00261 | Downregulation | -1.24368 | 4.05E-07 | 4.84E-05 |
| LINC00114 | Downregulation | -1.03379 | 2.33E-07 | 3.11E-05 |
| LINC00494 | Upregulation | 1.207072 | 1.36E-07 | 2.15E-05 |
| LINC00355 | Upregulation | 1.295757 | 0.000575 | 0.0163 |
| HOTAIR | Upregulation | 1.461387 | 4.59E-06 | 0.000385 |
| FAM41C | Upregulation | 2.14744 | 8.84E-15 | 5.47E-12 |
| RMST | Upregulation | 3.111766 | 2.81E-26 | 6.37E-23 |

**Table S5** Clinical covariates in the training and testing sets

| Covariates | Group | Total n=544 | Training set n=272 | Testing set n=272 | *P*-value |
|---|---|---|---|---|---|
| Survival time | | 1.97±0.09 | 1.94±0.13 | 2.00±0.11 | 0.722 |
| Vital status | Alive | 446(81.99%) | 226(83.09%) | 220(80.88%) | 0.799 |
| | Dead | 98(18.01%) | 46(16.91%) | 52(19.11%) | |
| Stage | I | 95(17.65%) | 43(15.99%) | 52(19.33%) | 0.631 |
| | II | 208(38.66%) | 99(36.80%) | 109(40.52%) | |
| | III | 149(27.70%) | 85(31.60%) | 64(23.79%) | |
| | IV | 86(15.99%) | 42(15.61%) | 44(13.36%) | |
| T stage | T1 | 17(3.12%) | 9(3.31%) | 8(2.94%) | 0.940 |
| | T2 | 93(17.10%) | 47(17.28%) | 46(16.91%) | |
| | T3 | 375(68.93%) | 182(66.91%) | 193(70.96%) | |
| | T4 | 59(10.85%) | 34(12.50%) | 25(9.19%) | |
| N stage | N0 | 316(58.09%) | 150(55.15%) | 166(61.03%) | 0.697 |
| | N1 | 129(23.71%) | 71(26.10%) | 58(21.32%) | |
| | N2 | 99(18.20%) | 51(18.75%) | 48(17.65%) | |
| M stage | M0 | 457(84.01%) | 230(84.56%) | 227(83.46%) | 0.940 |
| | M1 | 87(19.99%) | 42(15.44%) | 45(16.54%) | |
| Age | <=65 | 235(43.20%) | 118(43.38%) | 117(43.01%) | 0.996 |
| | >65 | 309(56.80%) | 154(56.62%) | 155(56.99%) | |
| Gender | Female | 257(47.24%) | 137(50.37%) | 120(44.12%) | 0.344 |
| | Male | 287(25.76%) | 135(49.63%) | 152(55.88%) | |

**Table S6** 3-lncRNA risk score model

| LncRNA | Coeffcient | Exp(coef) | Se(coef) | z | Multivariate p-value |
|---|---|---|---|---|---|
| LINC00114 | -0.2257 | 0.7979 | 0.0921 | -2.45 | 0.014 |
| LINC00261 | -0.1477 | 0.8627 | 0.0719 | -2.05 | 0.040 |
| HOTAIR | 0.1184 | 1.1257 | 0.0624 | 1.90 | 0.058 |

**Table S7** The top 200 mRNAs co-expressed with LINC00261 and HOTAIR

| lncRNA | mRNA |
|---|---|
| LINC00261 | FOXA2, ANG, FOXA3, FAM174B, HGD, KIAA1324, RAP1GAP, SERPINA1, MLPH, HNF4A, AGR2, FMO5, HEPACAM2, SLC43A1, B3GNT6, HPN, SH3BGRL2, GSTA1, RPH3AL, TFF1, SPDEF, STARD10, TCEA3, APOB, TFF3, APOA2, AMBP, MIA3, CREB3L1, CAPN9, PTPRN2, RAB26, TSPAN13, CACNA2D2, ST6GALNAC1, FAM149A, SPINK4, MTTP, REG4, GPRC5C, AQP3, AGR3, TTR, DDC, NEDD4L, MAGI1, C5, UNC13B, NAT6, MYO5C, MUC2, DNAJC12, CGNL1, ASRGL1, KCTD14, MARVELD2, TNFRSF11A, A1CF, VSIG2, ABCC6P1, ARSE, APOA1, GSTA4, FCGBP, MRAP2, C4BPB, CHDH, MGST2, WFDC2, HABP2, RAB17, CREB3L4, DNAJC22, IQGAP2, |

| | |
|---|---|
| | AKR1D1, TMEM56, GATA6, MBNL3, SORBS2, CHN2, SLC4A4, TPD52, ITLN1, HMGCS2, SMAD9, RBP4, ABCC6, SMCO4, ALDH3A2, GALNT8, SIDT1, CA8, TOX, SLC22A23, NOSTRIN, FOXP1, APOH, ALDH1A1, GJB1, NR3C2, CD302, RASSF6, SLC18A1, SERPIND1, GNE, PLEKHB1, MAOA, HIPK2, GMDS, PGM3, UGT2B11, MYRF, GP2, MCF2L, SMIM14, CYB5A, CRACR2A, RGN, PDXDC1, KLB, SYTL5, EPHX2, ALDH6A1, ST7, RORC, RASD1, FFAR4, IVD, HID1, KLK1, SEMA4G, ADH6, WNK4, CAMK2D, FZD5, TM9SF3, PTP4A1, SSTR1, ATP2A3, RNF128, F2, SCNN1A, AMT, AMDHD1, FAM107B, CRACR2B, GATA6-AS1, DNALI1, CTSE, VIL1, CPB2, EHHADH, SMLR1, PLXNA2, MYRIP, SPRED2, TMEM92, ANO1, KDELR2, GAS2, RAB27A, VTN, HSD17B2, GATA4, F5, DUSP4, PCCA, ECHDC2, MAML3, HNF1B, CRYM, NHSL1, MPC2, ATP2C2, ETFDH, JPH1, CADPS2, RASEF, APOC1, PON3, ABCD3, AGMAT, TSPAN8, COLCA2, UGT2B15, PAH, ATOH1, CLDN18, PRSS1, PROX1, NR0B2, SIDT2, GC, CMTM8, TMPRSS2, TMEM61, IL17RB, SLC27A3, CA2, PLD1 |
| HOTAIR | HOXC10, HOXC9, HOXC11, HOXC13, HOXC6, HOXC4, CACNB3, HOXA11, HOXA10, TRPS1, EMP2, CSAD, TANC1, DNAJC22, SMAD6, IQCE, CRNDE, HOXA13, SERTAD4, ZBTB41, TBX3, THOC2, SLC35A1, NFIA, NIPAL2, HOXA9, HOXB7, CREB3L4, KCTD15, RABEP2, SIM1, SIX1, S100A11, RHBDF1, TNRC18, CETN2, TTC30B, GTF2IRD1, SPATS2, ELF3, GATA2, VPS45, FAF2, SDC1, BAMBI, TFAP2A, TCEA3, KDELR2, HOXC5, GGPS1, STK3, ARHGEF12, TMTC3, BROX, PTPRK, LGALS8, GRTP1, PLA2R1, PLXNB2, NBPF3, OSR2, TCAF1, PON2, CDK19, HOXD10, KDM5B, MORC4, NOL3, SCYL3, SRD5A1, PIGM, CAMK2N1, PPIC, KIAA0895, ANTXR1, ZFHX3, SELENBP1, ARID5B, HOXB3, TEAD2, GPR137B, ELL3, FARP1, VDR, ZNF436, ATL3, EPPK1, TULP3, TCTEX1D2, LIMCH1, LRP11, PURB, GOPC, ENAH, PEX13, MKX, GABPB2, ANXA4, ABCC3, DCAF6, LRIG3, HOXD11, MEIS1, IGSF3, NAALADL2, ERGIC2, SLITRK6, TTC3, KCNK1, PEX11A, PIAS3, HOXB13, SMYD2, SLC30A5, GPRC5A, IFT22, SLC2A10, LURAP1L, IDH2, IRX5, IER5L, MBD6, IL13RA1, GPX8, ZNF32, PTK7, NFYB, TSPAN13, FKBP14, IGFBP5, RABGAP1, KNOP1, ID2, ATF7, NCOA3, SOX4, SYCP2, TEAD3, CTPS2, GLT8D2, GUK1, NUPR1, TMEM237, FAM149B1, ZFYVE19, CMAHP, ERGIC1, HFE, MIR4680, RAB27B, IRF2BP2, PDCD4, ARSD, SLC37A1, ZNF703, ERMAP, RNF187, UAP1, PDCD6, GABRE, PPFIBP2, PRKAA1, DOK4, FAM83H, DPY19L4, GAS1, IFT81, LINC00968, ZHX1, SEC63, RXRA, HSPB1, LRRC37A3, COL12A1, TWF1, CDS1, S100A11P1, EFNA4, PXDN, UBE2A, EPS8, DSTN, THBS3, PLXNA3, MAGED1, AJUBA, GGCT, THBS2, ROCK2, CRABP2, GLIS2, COL1A1, TRIM56, SEC16A, ANAPC7, TMEM106C, IFT43, TOB1, COL5A1, UBE2Q2 |
